# Two-Dimensional, Vision-Based Measurement for Experimental Characterization of Planar Compliant Mechanisms: A Critical Review and Uncertainty-Aware Framework

**DOI:** 10.3390/mi17091031

**Published:** 2026-08-29

**Authors:** Rohan R. Ozarkar, Nilesh P. Salunke, Prajitsen G. Damle, Shakeelur Raheman, Khursheed B. Ansari

**Affiliations:** 1 Department of Mechanical Engineering, R. C. Patel Institute of Technology, Shirpur 425405, India; 2 Department of Mechanical Engineering, SVKM’s NMIMS Global University, Dhule 424001, India; nilesh.salunke@svkm.ac.in; 3 Department of Mechanical Engineering, Shram Sadhna Bombay Trust’s College of Engineering and Technology, Jalgaon 425001, India; damle.prajitsen@sscoetjalgaon.ac.in; 4 Department of Applied Science and Humanity, SVKM’s NMIMS Global University, Dhule 424001, India; shakeelur.ateequrraheman@svkm.ac.in; 5 Department of Chemical Engineering, College of Engineering, King Khalid University, Abha 61421, Saudi Arabia

**Keywords:** 2D measurement, machine vision, camera calibration and digital image correlation, OpenCV, kinematic characterization, planar and compliant mechanisms

## Abstract

In planar compliant mechanisms, single-input dual-output (SIDO) displacement amplifiers driven by piezoelectric actuators are frequently used in precision positioning, micro/nano manipulation, and biomedical microdevices. Accurate experimental verification of these mechanisms remains challenging because traditional contact sensors can add excess stiffness and impact the structure’s normal behavior, and single-axis interferometers cannot measure multiple points simultaneously. In contrast, 2D vision-based measurement offers a non-contact alternative capable of capturing full planar motion and synchronized displacement tracking within a single image frame. This paper reviews the literature on camera calibration, homography-based planar reconstruction, sub-pixel edge extraction, vision-based characterization of compliant mechanisms, and benchmarking of vision systems against laser interferometers and coordinate measuring machines (CMMs). In the review, the SIDO-CDAM developed by Ozarkar et al. based on the Instantaneous Center Building Block (IC-BB) approach has been chosen as the target characterization system. The reviewed studies confirm that the major technical components needed for a high-precision 2D vision framework have been independently validated. However, there seems to be a lack of an integrated framework designed for synchronized dual-output SIDO-CDAM characterization. To overcome this gap, a seven-layer 2D vision-based characterization framework is proposed for scalable inspection of prototype-scale and MEMS-scale compliant mechanisms.

## 1. Introduction

Planar compliant mechanism systems, in which all elements are constrained to move in a single plane, are found in a variety of engineering domains, including robotic manipulators and automotive suspension systems, precision positioning stages and biomedical microdevices [1,2,3,4]. Accurate experimental characterization of such systems remains essential for validation of kinematic and dynamic models, determining the existence of manufacturing-induced deviations from the intended design, and enabling reliable iterative optimization. In compliant mechanisms, motion is obtained through elastic deformation rather than traditional rigid-body joints [5]. Therefore, the displacement, velocity, and force distribution in a planar mechanism must be measured with sufficient accuracy and spatial resolution to distinguish finite element analysis (FEA) predictions from actual physical behavior.

Conventional contact-based instruments such as linear variable differential transformers (LVDTs), rotary encoders, dial gauges, and strain gauges can achieve high measurement accuracy. However, they face several limitations when applied to compliant mechanisms. Any sensor (i.e., contact probe or strain gauge) physically attached to the mechanism provides stiffness and mass loading and thus affects the structural response to be measured. Further, a single sensor detects only one displacement component. The 2D output field (i.e., primary displacement along with parasitic lateral displacement) of a dual-output mechanism requires two sensors for characterization. Each of the individual sensors can add its own loading and installation error, indicating that their engagement is often complex and time-consuming. Sequential sensor measurements cannot capture the correlated and synchronized motion behavior of dual-output compliant systems. Moreover, contact instruments remain limited in measurement range and appear costly in terms of instrumentation and maintenance [5]. Therefore, contact-based measurement becomes less suitable for compliant mechanism characterization, especially at micrometer-scale displacement levels.

Recently, vision-based measurement systems have emerged as an effective non-contact alternative for planar motion analysis and precision metrology. A 2D vision-based measurement framework tackles many of the limitations of contact sensors. The motion of several tracking points in the same image frame can be recorded with a single calibrated camera, without disturbing the mechanism under study. Modern sub-pixel image processing algorithms allow the feature position to be determined with a precision much smaller than the native pixel resolution. The homography-based planar reconstruction allows an accurate conversion between image coordinates and real-world coordinates [6]. The primary advantage of vision-based systems comes from the capability of performing remote, simultaneous, and multi-point measurements of displacements without affecting the structural behavior of the system under study. These devices are therefore very well suited for experimental mechanics, structural monitoring, and compliant mechanism characterization.

This review is motivated by the specific mechanism, which is the Single-Input Dual-Output Compliant Displacement Amplification Mechanism (SIDO-CDAM) introduced by Ozarkar et al. [1]. The mechanism includes a monolithic planar compliant body that turns the small stroke of a single piezoelectric stack actuator into two simultaneous, symmetric amplified displacements at distinct output ports. The design utilizes the Instantaneous Center Building Block (IC-BB) synthesis method in which compliant dyad building blocks (CDBs) are systematically integrated to achieve the desired kinematic behavior. A structured two-stage multi-response optimization approach is employed to simultaneously maximize geometric advantage (GA) and minimize structural stiffness, followed by finite element validation of the optimized configuration. Since compliant displacement amplification mechanisms fundamentally follow energy conservation principles, an increase in geometric advantage (GA = $\delta_{out}$/$\delta_{in}$) is accompanied by a proportional reduction in output force [7]. Accurate measurement of GA in a fabricated prototype, therefore, requires a non-contact, simultaneous, multi-point characterization methodology capable of resolving both primary and parasitic displacements.

For the characterization framework considered in this review, three key experimental requirements arise directly from the SIDO-CDAM configuration, viz., (a) simultaneous tracking of the input point and both output ports within a single image frame (b) displacement measurement resolution better than 2 µm across the full operating rang and (c) validation of the measured results against a traceable reference instrument such as a laser interferometer or precision stage.

The 2 µm figure is proposed as a working target rather than a value derived from a reported FEA-experiment discrepancy for this specific prototype (no such experimental comparison has yet been published for the Ozarkar et al. [1] SIDO-CDAM). It represents approximately 0.3–4% of the 50–700 µm operating range and remains intended to resolve parasitic displacement components, typically an order of magnitude smaller than the primary amplified output, from measurement noise. This target is advised to be revisited once experimental FEA-vs-prototype discrepancy data become available.

To the best of the authors’ knowledge, a unified framework integrating camera calibration, planar homography, motion compensation, sub-pixel feature extraction, simultaneous dual-output tracking, and uncertainty-oriented validation for SIDO-CDAM characterization has not yet been systematically reported in the literature. The primary gap identified in existing studies is therefore integrative rather than purely technical.

The major contributions of this review are summarized as follows: (i) systematic review of vision-based measurement methodologies relevant to planar compliant mechanisms; (ii) critical comparison of precision metrology approaches used for compliant mechanism characterization; and (iii) proposal of a seven-layer 2D vision-based characterization framework specifically intended for SIDO-CDAM applications.

This paper is organized as follows. Section 2 reviews camera calibration and homography-based planar tracking methods. Section 3 discusses sub-pixel edge detection techniques and their achievable measurement accuracy. Section 4 reviews vision-based characterization approaches for compliant mechanisms. Section 5 examines compliant displacement amplification mechanisms and associated experimental measurement challenges. Section 6 compares vision-based systems with other approaches for precision metrology and identifies the major research gaps in the current literature. Section 7 presents the proposed seven-layer 2D vision-based characterization framework and the uncertainty-oriented methodology. Finally, Section 8 concludes with a summary of the major findings and directions in future research.

## 2. Camera Calibration and Homography-Based Planar Tracking

### 2.1. Camera Calibration Basics

Camera calibration refers to the process of estimating the intrinsic and extrinsic parameters, describing how a camera observes the physical world. These parameters include the focal length, the principal point, the lens distortion coefficients, the position, and orientation of the camera with respect to the measurement plane. Zhang’s checkerboard-based calibration method [8] is preferred, as it requires few images of a planar calibration target captured from different angles and remains easy to implement in computer vision libraries such as OpenCV. For the characterization of the planar compliant mechanism, accurate lens distortion correction is specifically crucial, as uncorrected distortion can cause considerable position errors at the boundary of the image that affect the accuracy of displacement measurement. This effect becomes more critical when micrometer-level displacement resolution is required. The application of telecentric optics can significantly minimize the perspective distortion and magnification variation within the field of view, thereby improving the measurement uniformity.

**Figure 1:**
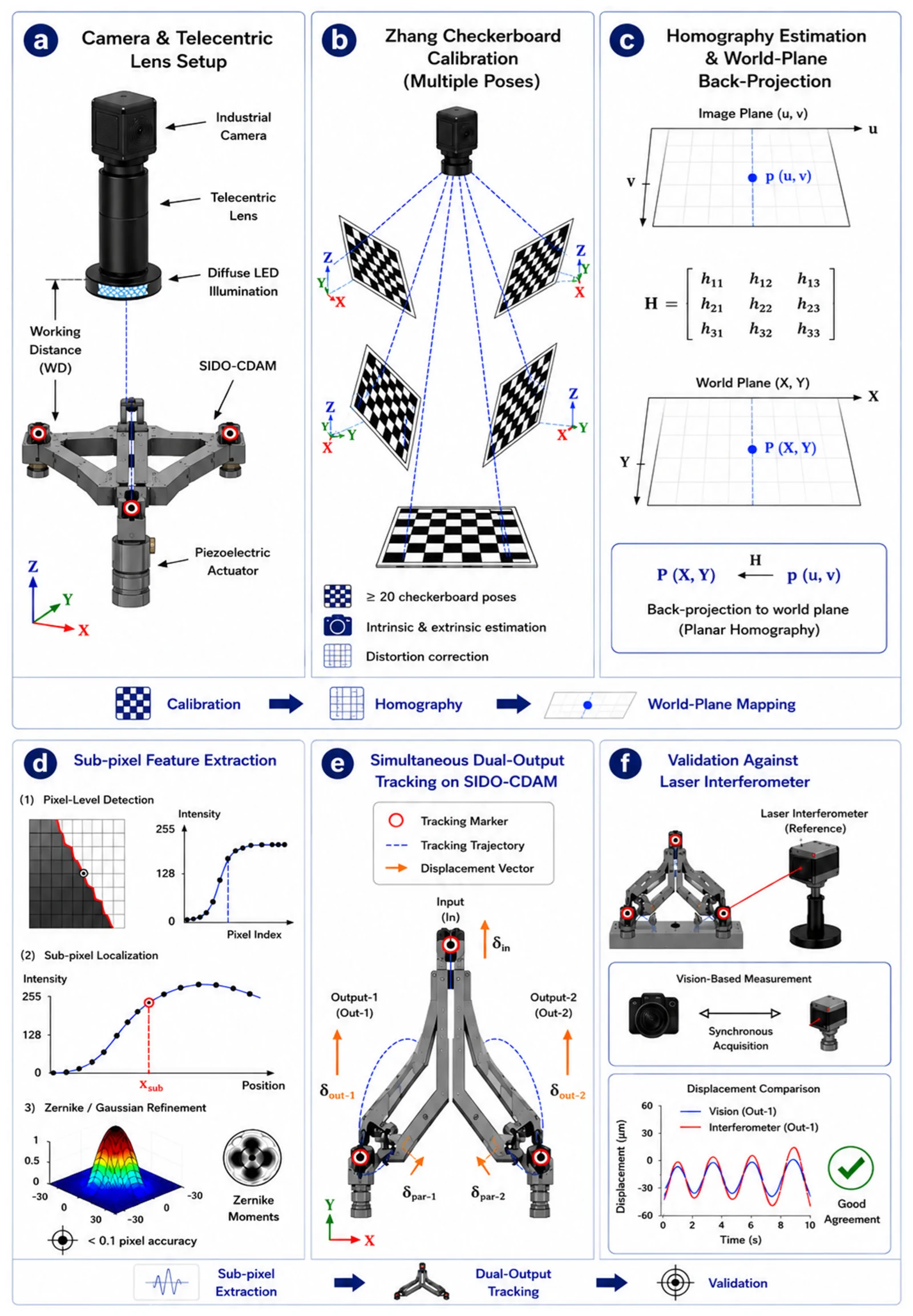
Pipeline of the 2D vision-based measurement framework. (**a**) Camera and telecentric lens arrangement; (**b**) Zhang checkerboard calibration using various poses; (**c**) homography estimation and world-plane back-projection; (**d**) simultaneous dual-output marker tracking on a CDAM; (**e**) Simultaneous tracking on SIDO-CDAM; (**f**) validation against laser interferometry.

Arellano-González et al. [9] investigated the performance of homogeneous and non-homogeneous Direct Linear Transformation (DLT) calibration methods for the motion tracking of planar mechanisms. They reported comparable findings for both methods under controlled laboratory conditions and showed that with appropriate calibration procedures, reliable planar motion tracking can be achieved using low-cost machine vision hardware. However, such findings should be read as demonstrating reliable relative motion tracking under the controlled conditions and not as evidence that low-cost hardware alone meets the targeted sub-2 µm absolute resolution. Arellano-González et al. [9] failed to report displacement resolution at that specific level. The pixel-to-distance scale factor achieved with non-telecentric low-cost optics is generally less uniform across the field of view than with telecentric optics. The specific benefit telecentric optics adds over affordable non-telecentric hardware is a spatially uniform magnification, meaning the pixel-to-mm conversion factor does not vary with target position or working-distance fluctuation within the depth of field. This uniformity allows the scale-factor and homography methods to converge (cf. Section 2.2) and remains the basis for adopting telecentric optics as a framework requirement, despite [9]’s finding that low-cost non-telecentric hardware suffices for reliable relative tracking in less demanding applications. Figure 1 presents the typical workflow of a 2D vision-based characterization system, which includes camera calibration, homography estimation, planar reconstruction, and simultaneous multi-point tracking.

### 2.2. Homography-Translating Image Pixels to Real-World Coordinates

Planar motion can be reconstructed using a homography transformation once the camera is calibrated. In the case of strictly planar motion, the projective mapping between the image coordinates and corresponding real-world coordinates on the measurement plane can be obtained by a homography matrix H. Homography-based reconstruction compensates for perspective effects and non-uniform magnification across the image, which the simple pixel-to-millimeter scale-factor method does not. This distinction matters primarily when the sensor plane remains non-parallel to the measurement plane or when the optics are not telecentric. Under ideal telecentric imaging with the sensor plane parallel to the measurement plane, the scale-factor method is mathematically equivalent to a homography with zero perspective components; hence, the two approaches converge in that specific configuration. Homography reconstruction retains an advantage primarily as a safeguard against residual misalignment and non-ideal optics.

Wu et al. [10] recommended a homography characteristic matrix method in which the homography matrix is decomposed using singular value decomposition (SVD) to directly recover translational and rotational motion components. The method was used on a three-degree-of-freedom robotic stage and provided higher accuracy and better repeatability than conventional methods based on scale factor. The scale-factor method assumed a constant ratio of pixels to distance throughout the image and, hence, is not accurate for larger fields of view or non-telocentric optical systems. Chen et al. [11] proposed a high-resolution stage measurement based on a 2D-DFT phase estimation approach using grating patterns with a nanometer-scale measurement resolution. Another study by Chen et al. [12] showed long-range planar position detection by machine vision with high linearity (0.04%) over broad travel ranges (i.e., 40 mm). These studies collectively indicated the feasibility of using homography-based vision systems for both short-range high-precision measurement and larger-scale planar motion tracking applications.

These results should not be read as directly comparable, and the terms should not be used interchangeably: Chen et al. [11] report resolution (the smallest detectable displacement increment, 3.5–8 nm), Chen et al. [12] report linearity (how proportionally the measured output tracks true displacement across a travel range, 0.04%, which at their 40 mm range corresponds to roughly ±16 µm of potential non-proportionality, not nanometer-scale resolution), and Wu et al. [10] report accuracy and repeatability (agreement with a known reference and consistency across repeated trials) without a stated resolution figure. For the SIDO-CDAM’s 50–700 µm operating range, the relevant target from Section 1 is displacement resolution and accuracy at the sub-2 µm level over that range, which is closest in kind to the results of Chen et al. [11] and Wu et al. [10], while Chen et al. [12]’s linearity figure characterizes a different property (large-range proportionality) that is less directly applicable at the SIDO-CDAM’s smaller travel range.

### 2.3. Compensation for Camera Motion and Environmental Disturbances

One practical problem in vision measurement at the laboratory scale is the unintentional motion of the camera due to environmental vibration, disturbance of the optical table, or thermal drift. Even small camera movements in the micrometer range can appear as incorrect structural displacement during compliant mechanism characterization. Since compliant displacement amplification mechanisms typically operate in displacement ranges of only a few hundred micrometers, compensation of the camera motion remains important for reliable experimental measurement.

To address this problem, Jiao et al. [13] proposed a vision-based motion correction strategy based on fixed reference markers on the stationary base structure. The apparent displacement of the reference markers in each image frame is used to estimate unintended camera motion, and it is removed from the measured displacement of the moving structure. It is a combination of Random Sample Consensus (RANSAC) for outlier rejection and Efficient Second-order Minimization (ESM) for robust homography estimation and sub-pixel tracking performance.

For SIDO-CDAM characterization, several (4–6) reference markers can be positioned on the fixed base plate outside the active deformation region of the mechanism. This count is proposed rather than experimentally optimized: four points are the minimum required to constrain a planar homography, and the additional one to two points provide redundancy for RANSAC-based outlier rejection [13] when a marker is obstructed, poorly illuminated, or affected by local vibration in a given frame. Marker placement affects the resulting drift-compensation accuracy. Markers clustered near the mechanism center are more sensitive to rotational drift, while markers spread toward the corners of the field of view better constrain both translational and rotational components of unintended camera motion, at the cost of being farther from the tracked output ports and thus more exposed to any spatial non-uniformity in lens distortion correction. The measured motion of these markers can then be used for drift compensation on a frame-by-frame basis to minimize the effect of environmental vibration and thermal instability on displacement estimation. As the target measurement uncertainty approaches the sub-pixel range, this motion compensation becomes ever more important.

Table 1 summarizes representative studies from the reviewed literature on camera calibration, planar reconstruction, and accuracy of vision-based displacement measurement. The studies differ in field of view, optical magnification, working distance, and reference instrument, and report different metrics (calibration error, accuracy, repeatability). The values are therefore reported as published, for orientation, and should not be read as directly comparable to one another.

## 3. Sub-Pixel Edge Detection

### 3.1. The Resolution Gap and Sub-Pixel Methods

In machine vision systems, the native spatial resolution is fundamentally limited by the camera sensor and optical magnification. For example, in the case of an 80 mm × 80 mm compliant mechanism imaged with a conventional 4 MP industrial camera, one pixel corresponds to about 32 μm of real distance. Such a resolution remains insufficient for experimental characterization of CDAM.

This limitation is overcome by sub-pixel edge detection algorithms, which estimate the edge location at a fraction of a pixel by modelling the local intensity distribution around the edge region. Instead of treating the edge as a discrete pixel boundary, the algorithm interpolates the gray-scale intensity transition to determine a more precise edge position. Under suitable illumination and imaging conditions, the localization precision of modern sub-pixel approaches can reach 0.05–0.10 pixels, corresponding to a micrometer-level physical resolution depending on the optical magnification and field of view [16].

The achievable accuracy of a sub-pixel measurement system depends not only on the algorithm itself, but also on practical imaging parameters, such as signal-to-noise ratio, stability of illumination, optical distortion, quality of the marker, and mechanical vibration. Hence, algorithm selection must be considered in the context of the overall imaging configuration rather than as a stand-alone image processing step.

### 3.2. Comparative Assessment of Sub-Pixel Edge Detection Methods

#### 3.2.1. Coarse-to-Fine Hybrid Methods

Among the reviewed studies, coarse-to-fine hybrid methods appear to be especially suitable for compliant mechanism characterization since they provide a trade-off between computational efficiency and measurement accuracy. Xie et al. [16] proposed a two-stage strategy for edge extraction. Firstly, a fast Roberts operator is used to search for the rough edge location. Then, an improved Zernike moment calculation is used to refine the edge location to sub-pixel accuracy. Otsu’s method sets the intensity threshold automatically. The hybrid strategy was shown to have increased precision and better computational efficiency compared with traditional Zernike-only implementations. This method is especially suitable for CDAM because the output markers and flexure boundaries tend to be smooth, linear, or mildly curved geometries produced by wire-EDM or precision CNC machining.

#### 3.2.2. Zernike Moment and Gray-Level Moment Methods

Zernike moment methods estimate the edge position and orientation by fitting orthogonal polynomial functions to the local image intensity distribution. One advantage of this approach is that edge location and directional information can be acquired simultaneously, which is useful for tracking curved flexure boundaries and components of rotational motion.

Guo et al. [17] used a sub-pixel method based on the Zernike matrix for dimension measurement of flange disc parts and showed good agreement with coordinate measuring machine (CMM) measurements. Ding et al. [18] further improved the Zernike approach for curved-edge extraction, relevant to the compliant mechanisms employing circular notch hinges or curved flexure geometries. Dynamic displacement tracking has also been explored using Gray-Level Moment (GLM) methods. Hagara et al. [19] demonstrated that edge localization based on the GLM model can successfully track displacements of less than one pixel for partially blurred imaging conditions. This capability remains particularly important for compliant mechanism testing near resonance frequencies, where motion blur can degrade the performance of conventional edge extraction.

#### 3.2.3. Gaussian Integral Method

Gaussian-based edge modelling methods approximate the image intensity transition across an edge using the integral form of a Gaussian function. Duan et al. [20] used a Gaussian integral fitting (least-squares) method for the precision measurement of gear tooth profiles, and the maximum measurement error was about 1.9 μm. Compared with higher-order polynomial approaches, the Gaussian fitting approaches generally have a lower computational complexity with good accuracy for relatively smooth edge geometry. However, their performance can degrade for very irregular or noisy edge profiles. Hence, Gaussian approaches may be more suitable for straight or slightly curved marker boundaries than complex flexure contours for the characterization of compliant mechanisms.

#### 3.2.4. Hessian-Based Methods (Canny–Steger)

Hessian-based methods determine the edge locations by using second-order image intensity derivatives. Cheng et al. [21] reported 3 μm accuracy and 2 μm repeatability on shaft parts by coupling Canny edge detection with Steger’s Hessian-based method. A benefit of Hessian-based methods is their ability to accurately detect edges along regions of maximum intensity curvature, and hence are useful for curved flexure hinges and circular notch geometries, which are common in compliant mechanisms. However, these methods are usually more computationally expensive and can be sensitive to image noise if the illumination conditions are not well controlled.

#### 3.2.5. Deep Learning-Based Sub-Pixel Methods

Recent work has studied the application of deep learning for sub-pixel edge localization. A CNN-based approach for sub-pixel line-edge angle detection was proposed by Pang et al. [22], which improved accuracy over traditional methods for inclined edges. Deep learning approaches offer potential benefits for illumination variation, surface texture irregularities, and complex edge conditions. However, they often require extensive labelled training datasets and high computational resources. In addition, their measurement uncertainty characteristics are often less interpretable than analytical edge models. At the current stage, the adoption of deep learning-based sub-pixel methods in the precision CDAM characterization is still at an early stage and not yet recommended for primary measurement use.

### 3.3. Practical Factors Affecting Sub-Pixel Measurement Accuracy

In addition to algorithm selection, various practical imaging factors strongly influence the achievable measurement accuracy of a vision-based characterization system.

- Illumination Conditions: The surface finish of the wire-EDM or CNC-machined planar mechanisms is typically semi-reflective, which results in illumination-dependent intensity variation and unstable edge localization. Diffuse coaxial LED illumination is generally preferred because it minimizes specular reflection and provides consistent edge contrast throughout the mechanism’s motion.
- Optical Configuration: Telecentric lenses are most suitable for precision planar measurement as they offer almost uniform magnification in the field of view and significantly minimize perspective distortion. This leads to a stable pixel-to-distance relationship over the entire measurement space, which simplifies calibration and improves reconstruction accuracy.
- Thermal Drift and Sensor Stability: Industrial CMOS sensors may exhibit thermal drift during the initial operation. Proper warm-up time before measurement can alleviate image instability caused by drift. For long-term experiments, this helps keep sub-pixel measurements consistent when used with reference marker-based drift correction.
- Marker design: The quality of edge extraction is closely linked to the marker contrast and geometry. For prototype-scale compliant mechanisms, circular and square markers, laser-engraved or ink-printed, usually provide sufficient contrast for reliable sub-pixel tracking without significantly impacting structural performance. Optical tracking of MEMS-scale mechanisms may require lithographically patterned markers or etched surface features.

These practical considerations demonstrate that micrometer-scale experimental characterization relies on the combined performance of optics, illumination, calibration, mechanical stability, and image-processing algorithms, instead of relying solely on sub-pixel algorithms. None of the reviewed studies numerically quantify the illumination, thermal drift, or marker-design-related contributions to measurement uncertainty for a configuration comparable to the proposed framework; these are treated as open items in the uncertainty budget presented. Table 2 summarizes representative sub-pixel edge detection methods and the reported measurement performance in the literature reviewed.

## 4. Vision-Based Experimental Characterization of CDAM

### 4.1. Need for Non-Contact Characterization in CDAMs

Compliant mechanisms produce motion by utilizing elastic deformation of flexure members rather than traditional rigid-body joints. Therefore, their structural response is highly sensitive to the imposed constraints and loading conditions. Inequalities in finite element predictions and experimentally measured results may occur during experimental validation because the contact force applied by a displacement sensor may alter the mechanism’s deformation characteristics. This issue becomes more crucial for compliant displacement amplification mechanisms where large output displacement is obtained by distributed elastic deformation. Accurate experimental evaluation, therefore, requires measurement of input displacement, amplified output displacement, and parasitic motion simultaneously while affecting the mechanism itself as minimally as possible. Conventional contact-based instruments are often not suitable for this purpose as they typically tend to measure only a single displacement component. This may influence the effective stiffness of the compliant structure under test.

Many vision-based measurement systems overcome these limitations, enabling remote, noncontact, and multi-point displacement tracking. With a single calibrated camera, it is feasible to monitor multiple locations on the compliant structure simultaneously in the same image frame. This feature is especially beneficial for dual-output compliant mechanisms where simultaneous motion of multiple output ports needs to be experimentally validated.

### 4.2. Microscopic Vision for MEMS-Scale Mechanisms

Yao et al. [23] propose a microscopic vision system based on multi-scale Lucas-Kanade optical flow on high-magnification image sequences for a compliant nano-positioning stage. The system achieved an absolute measurement accuracy of 0.06 µm when validated against a laser interferometer, which is among the highest accuracies reported for vision-based characterization of compliant mechanisms in the literature reviewed. Similarly, Su et al. [24] used particle swarm optimization (PSO) combined with successive three-step search (S-TSS) template matching to a three-degree-of-freedom compliant micro-stage and obtained sub-micrometer measurement accuracy with high computational efficiency. These studies together demonstrate the capability of microscopic vision systems for precision characterization of MEMS-scale compliant mechanisms.

For the prototype-scale SIDO-CDAM developed by Ozarkar et al. [1], microscopic optics are not required, as the mechanism dimensions are significantly larger, with output ports separated by several tens of millimeters. The working displacement range of the mechanism is about 50–700 µm. A standard telecentric lens with a working distance of 100–200 mm can typically achieve 2–5 µm measurement accuracy, which is enough to resolve the FEA discrepancy observed experimentally in the operating range of the mechanism. This observation suggests the same general vision-based characterization approach using camera calibration, homography reconstruction, sub-pixel feature extraction, and synchronized tracking is applicable at both macro-scale and MEMS-scale, as the same four algorithmic steps appear in both the Yao et al. [23] microscopic implementation and the proposed prototype-scale framework.

It should not be claimed that only the optical magnification changes: moving from prototype to MEMS scale also requires changes in marker fabrication (lithographically patterned or etched features or laser-engraved markers), illumination requirements (higher magnification narrows depth of field and reduces working distance, depth of field, and vibration tolerances), and calibration procedure (microscope-based calibration targets differ from macroscopic checkerboards). Therefore, transfer of the framework to a MEMS-scale SIDO-CDAM would require suitable re-specification of these aspects rather than simple rescaling.

### 4.3. Force–Compliance Testing Using Vision Systems

Hricko and Havlík [25] proposed a practical experimental procedure called ‘vision-way testing’, in which loads are applied incrementally, and images are captured at each loading step. The displacement was gathered from the images to plot the force-compliance curve. The method captures actual flexure behavior, including nonlinear deformation effects and geometric imperfections, which may not be fully captured by finite element models assuming ideal beam behavior. The methodology is directly applicable to the characterization of SIDO-CDAM. For example, the voltage of the piezoelectric actuator can be increased stepwise from 0 to 100 V, and one image can be acquired at each loading step. The measured geometric advantage is then calculated as a continuous function of actuator voltage from the tracked input and output positions.

### 4.4. Full-Pose Vision for Kinematic Calibration

Renaud et al. [26] showed that the full kinematic calibration of a parallel robotic mechanism can be achieved with a single camera instead of precise physical calibration targets. Position and orientation full-pose vision measurements of the end-effector allow identification of optimal kinematic calibration models without high-precision physical targets. Their analysis of identifiability shows which parameters of the mechanisms can be identified from the image data only. The response for planar mechanisms includes GA, parasitic displacement ratio, and positions of output ports. Hence, a properly calibrated vision system provides users with a complete kinematic characterization of the SIDO-CDAM, without the need for additional displacement sensors.

### 4.5. Stereo Vision and Digital Image Correlation

Fuentes-Juvera et al. [27] implemented a low-cost stereo vision approach for kinematic validation of a 3D compliant gripper mechanism, reporting errors below 10% compared with FEA predictions. For strictly planar CDAMs, stereo calibration complexity and sensitivity to baseline misalignment outweigh the benefits of the third dimension—single-camera planar homography is both simpler and more accurate for this application.

Sutton et al. [6] presented a comprehensive review of Digital Image Correlation (DIC), a full-field displacement measurement technique based on random speckle pattern tracking. Commercial DIC systems such as Aramis and Vic-2D are widely used in experimental mechanics and material testing. However, several limitations arise when these systems are applied to miniaturized compliant mechanisms: (a) difficulty in applying high-quality speckle patterns to sub-millimeter flexure surfaces without affecting structural behavior; (b) high computational cost limiting real-time implementation; and (c) dependence on high-magnification optics that reduce the available field of view. For planar CDAM characterization, marker-based homography tracking therefore represents a more practical and computationally efficient solution.

For quasi-static characterization, such as piezoelectric voltage ramp testing below 1 Hz, a standard industrial camera operating at 25–60 fps is sufficient because the mechanism remains effectively stationary during image acquisition. The trade-off between imaging bandwidth, illumination intensity, and sub-pixel measurement accuracy remains insufficiently explored in existing CDAM characterization literature and represents a potential direction for future investigation.

### 4.6. Comparative Assessment of Approaches for SIDO-CDAM Characterization

Section 4.2, Section 4.3, Section 4.4 and Section 4.5 describe four distinct approaches: microscopic vision, incremental force-compliance testing, full-pose kinematic calibration, and stereo vision/DIC; each validated in a different experimental context. Table 3 compares them directly against the specific requirements of SIDO-CDAM characterization identified in Section 1, such as simultaneous input/output tracking, sub-2 µm resolution, and traceable validation.

The incremental force-compliance protocol [25] is judged the most suitable actuation procedure for the SIDO-CDAM framework because it requires no specialized optics and integrates directly with voltage-ramp testing (Section 4.3). Microscopic vision [23,24] is the most accurate approach demonstrated in the reviewed literature but is over-specified for the prototype-scale system and appropriate only for a future MEMS-scale variant (Section 4.2). Stereo vision and DIC are judged less suitable because the SIDO-CDAM is a planar mechanism, so their additional out-of-plane or full-field recovery capability is not needed, and their added complexity does not provide a corresponding accuracy benefit for this application. If out-of-plane measurement becomes necessary, the suitability of these approaches should be reconsidered.

## 5. Compliant Displacement Amplification Mechanisms: Design and the Measurement Problem

CDAMs are employed to transform the small input displacement of a piezoelectric actuator into a larger usable output displacement through elastic deformation of flexure members. Various amplification topologies have been reported in the literature, including lever-type, bridge-type, Scott-Russell, toggle-based, and hybrid compliant mechanisms [28,29,30,31]. The amplification performance of these mechanisms is commonly evaluated using geometric advantage, which is defined as the ratio of output displacement to input displacement. Other important design considerations, other than amplification capability, include output stiffness, parasitic displacement, stress concentration, dynamic response, and manufacturability.

**Figure 2:**
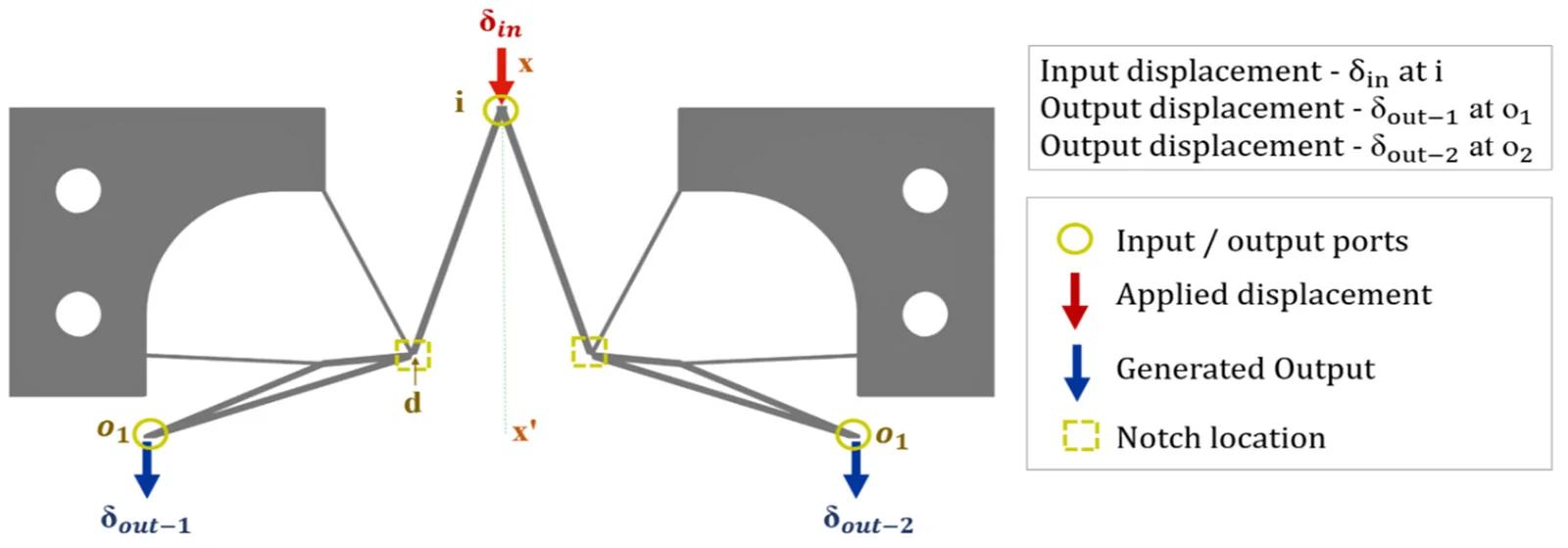
Schematic of a single-input port, two symmetric output ports (left and right) and a notch at d and d’ of SIDO-CDAM (Ozarkar et al. [1]). The same topology applies at prototype scale (mm) and miniaturized MEMS scale (µm). The vision framework is adjusted via lens magnification selection.

The mechanism of Ozarkar et al. [1], as shown in Figure 2, is based on a dual building block topology with dual symmetric output ports (Single Input Dual Output configuration). For a meaningful experimental validation of such a mechanism, the following quantities have to be measured simultaneously: (a) input displacement obtained from the piezoelectric actuator, (b) amplified displacement at output port 1; (c) amplified displacement at output port 2; (d) parasitic displacement components at both output ports, and (e) symmetry of motion between the two output branches.

A 2D vision-based framework is particularly appropriate for this application as all measurement points can be captured within a single synchronized image frame using a common coordinate system. Compared with sequential contact measurement approaches, simultaneous optical tracking synchronizes both output ports and limits the structural disturbance during testing.

The component accuracies reported in Section 2, Section 3 and Section 4 were each validated independently, on different hardware and under different test conditions than the SIDO-CDAM prototype. Independently validated component accuracies do not guarantee that the same accuracy translates to the integrated measurement chain, as uncertainties propagate and may accumulate. Section 7 applies a root-sum-square combination, under the standard assumption of uncorrelated sources, to quantify this accumulation for the present case rather than treating the individual literature values as directly applicable to the integrated system.

## 6. Benchmarking of Vision-Based Systems with Alternative Precision Metrology Techniques

Cui et al. [32] reviewed the accuracy hierarchy in precision displacement metrology, including laser interferometry (LI, sub-nanometre), grating interferometry (GI, approximately 1 nm), and time-grating sensors (TGS, approximately 1 nm). In comparison, vision-based systems typically achieve measurement accuracy in the range of 0.06–5 µm. Although laser interferometers provide substantially higher absolute accuracy than vision systems, they generally measure displacement only along a single axis at a time. A SIDO-CDAM with two output ports, for simultaneous measurement of six displacement quantities (X and Y displacements at three tracking locations), would require multiple independently aligned laser heads, making the setup experimentally complex and impractical for compact laboratory-scale compliant mechanisms. A single calibrated camera, however, can simultaneously capture all in-plane displacement components within the same image frame. Yao et al. [23] directly validated a microscopic vision system against a laser interferometer. The microscopic vision system was less accurate than the laser interferometer on the primary axis, but it simultaneously measured the transverse (parasitic) motion that could not be captured using a single-axis interferometer alone. Clark et al. [33] demonstrated an alternative indirect metrology approach in which a passive compliant mechanism converted 3D motion into three measurable 1D displacements. The method achieved near-interferometric resolution but required the fabrication of an additional precision mechanism specifically designed for measurement. Such an approach becomes impractical when compliant mechanism geometries change frequently during iterative design optimization.

Strain gauges [34] provide continuous, high-bandwidth displacement feedback but introduce stiffness loading. For miniaturized compliant mechanisms with flexure thicknesses in the range of 0.2–0.5 mm and overall stiffness between 1–10 N/µm, a typical foil gauge rosette can change the dynamics and resonance behavior of the mechanism. The vision-based characterization completely avoids this loading and is not subject to any mechanical perturbation to the system under study. Coordinate Measuring Machines (CMMs) provide traceable 3D dimensional metrology with sub-micrometer accuracy. However, CMMs require contact probing, are generally limited to quasi-static measurements, and cannot capture the dynamic force-displacement behavior during the actuation of the mechanism. Nogueira et al. [15] validated machine vision measurements against CMM measurements and observed a mean dimensional error of about 0.008 mm, which indicates that vision systems can provide CMM-equivalent accuracy for static planar dimensional measurements under suitable calibration conditions.

Kim et al. [35] demonstrated a hybrid interferometric and angular sensing system for 2D stage evaluation, achieving approximately 40 nm straightness uncertainty and angular accuracy near 0.14″. Traxler et al. [36] compared four optical inline measurement technologies, including stereo vision, photometric stereo, laser triangulation, and structured light systems. Their study reported lateral resolution in the range of 50–200 µm with temporal noise between 0.1–1 µm depending on the sensing configuration. For CDAM characterization, structured light and photometric stereo approaches may provide improved sensitivity to out-of-plane deformation and flexure buckling effects. However, these methods require substantially more complex optical calibration and illumination control compared with planar homography-based tracking. For strictly planar SIDO-CDAM characterization, single-camera 2D vision systems therefore provide a more practical balance between measurement accuracy, implementation simplicity, and simultaneous multi-point tracking capability. Table 4 summarizes the major advantages and limitations of different measurement approaches for SIDO-CDAM characterization.

The reviewed literature confirms that all the individual components required for a capable 2D vision-based characterization framework already exist and have been independently validated. Zhang’s checkerboard calibration, combined with homography characteristic matrix decomposition and singular value decomposition (SVD), achieves sub-pixel planar tracking accuracy [10]. Zernike hybrid sub-pixel extraction methods achieve measurement uncertainty in the range of 1–3 µm [16]. RANSAC-ESM homography tracking successfully compensates for camera vibration and laboratory drift [13]. Microscopic vision systems achieve 0.06 µm measurement accuracy for MEMS-scale compliant stages, while optical measurement of force–compliance behavior has also been experimentally demonstrated [23,25]. Vision systems have been shown to provide laser-tracker-equivalent workspace characterization [37] and CMM-equivalent dimensional measurement accuracy for planar components [14].

However, so far, no reported work has integrated all of these individual techniques into a single experimentally validated framework specifically intended for dual-output compliant displacement amplification mechanisms (SIDO-CDAMs). The primary research gap identified in the literature is therefore integrative rather than purely technical.

The individual layers of the proposed framework (optics, illumination, calibration, motion correction, feature extraction, tracking, validation) are each independently established in the reviewed literature. The specific contributions of the proposed framework are: (a) a synchronized dual-output tracking layer (Layer 6) that captures the input point and both output ports of a SIDO-CDAM within a single frame, absent from the reviewed single-point and single-axis studies; (b) a defined Motion Symmetry Index, MSI = (|δ_out-1 −_ δ_out-2_|)/δ_out,nominal_ and (c) a literature-grounded ISO-GUM uncertainty-budget methodology (Section 7) that propagates component-level uncertainties from the cited literature into a system-level estimate specific to this measurement chain, rather than citing component accuracies in isolation. These contributions constitute a proposed methodology and design target rather than an experimentally demonstrated result. Table 5 summarizes the major research gaps identified from the reviewed studies together with their implications for SIDO-CDAM characterization.

## 7. Proposed Seven-Layer 2D Vision-Based Characterization Framework

**Figure 3:**
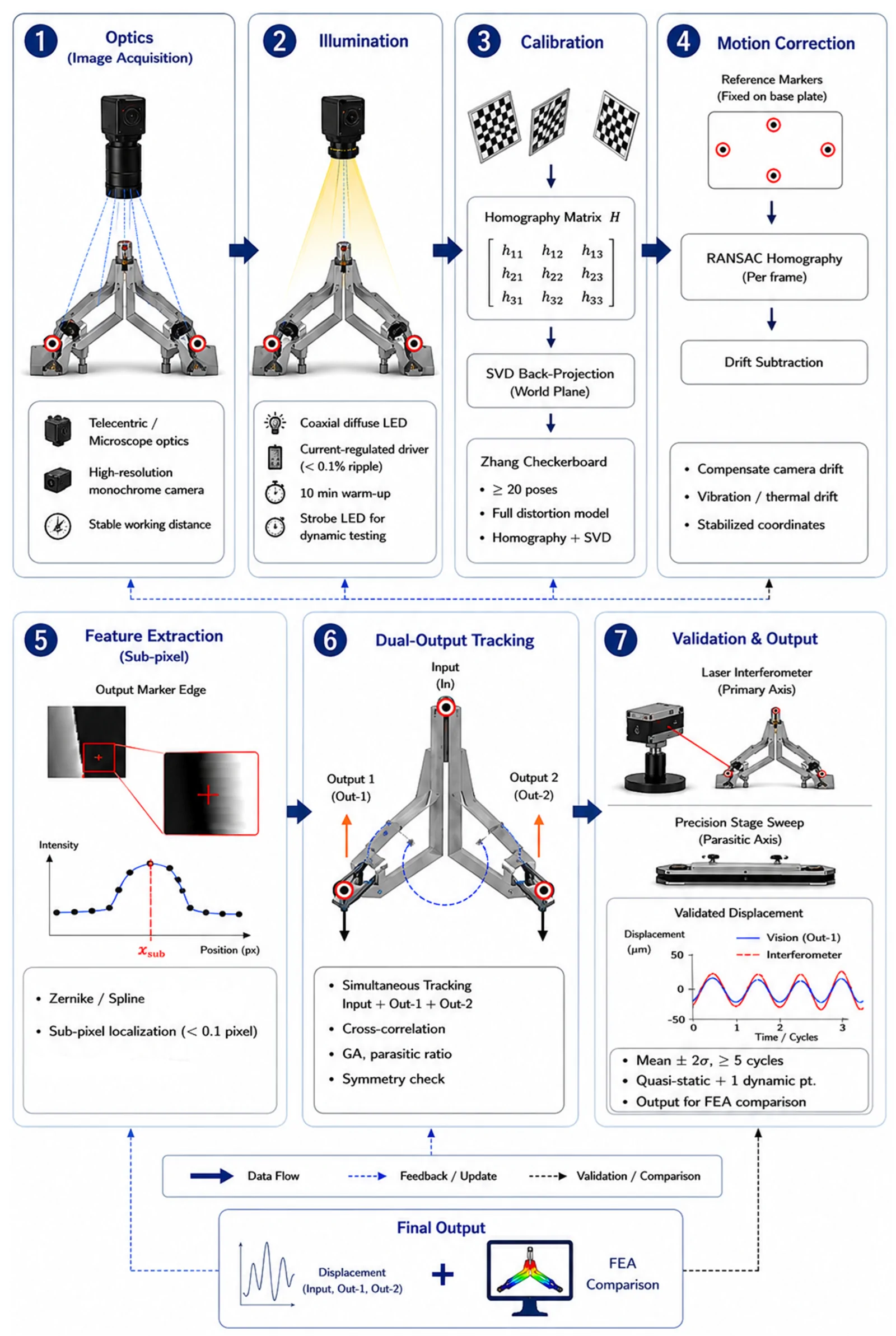
Architecture of the proposed seven-layer framework. Data flow from raw image acquisition to calibration, motion correction, sub-pixel extraction, dual-output tracking, validated displacement output, and FEA comparison.

The research gaps identified from the literature review motivate us to propose a seven-layer 2D vision-based characterization framework for experimental validation of SIDO planar CDAMs. The framework combines camera calibration, planar homography reconstruction, sub-pixel displacement extraction, motion compensation, synchronized multi-point tracking, and uncertainty-oriented validation in a unique experimental methodology. The proposed framework is mainly aimed at designing prototype-scale planar compliant mechanisms actuated by piezoelectric stacks. But the same method applies to MEMS-scale compliant mechanisms with necessary modifications in optical magnification and imaging setup. Figure 3 illustrates the general structure of the presented seven-layer framework, and Table 6 gives the corresponding layer-by-layer specification; the two are ordered identically. For prototype-scale SIDO-CDAM characterization, a 12–20 MP monochrome camera is usually enough for micrometer-scale displacement measurement. The telecentric lens reduces the perspective distortion and ensures that the magnification is almost constant in the measurement region (Layer 1: Optics). The second layer provides coaxial diffuse LED illumination driven by a current-regulated source to minimize specular reflection and maintain consistent edge contrast throughout the mechanism’s motion, with a strobe option for dynamic testing near resonance (Layer 2: Illumination). The third layer performs the intrinsic and extrinsic calibration using Zhang’s checkerboard calibration method together with planar homography reconstruction using reference markers mounted on the fixed base structure. The calibrated parameters generate the pixel-to-world coordinate transformation, compensate for lens distortion effects, and express the reconstructed coordinates of the moving output ports in the stationary base frame, enabling synchronized evaluation of the amplified displacement, parasitic displacement, and output symmetry (Layer 3: Calibration). The fourth layer compensates for environmental vibration, thermal drift, and unintentional camera movement. The measured drift is compensated using Random Sample Consensus (RANSAC)-assisted homography compensation by simultaneously tracking the stationary reference markers and the markers on the moving mechanism (Layer 4: Motion correction). The fifth layer uses a hybrid Zernike-based edge extraction for sub-pixel feature localization. Fiducial markers located near the actuator input and output ports are tracked in the image sequence. In general, under appropriate imaging conditions, localization accuracy of about 0.05–0.10 pixels can be achieved (Layer 5: Feature extraction). The sixth layer implements multi-point actuator input, dual-output ports, and reference-marker tracking synchronized in the same image frame. Simultaneous measurement enables direct evaluation of geometric advantage, parasitic displacement, and coupled deformation behavior and motion symmetry (Layer 6: Dual-output tracking).

The final layer (Layer 7) performs uncertainty estimation using International Organization for Standardization—Guide to the Expression of Uncertainty in Measurement (ISO-GUM)-based root-sum-square (RSS) propagation of the component uncertainties associated with Layers 1–6 [38]. Table 7 assembles this budget from the values reported in the reviewed literature at the ~20 µm/pixel spatial scale used in the Section 7.1 illustrative estimate (12 MP camera, 1× telecentric magnification). For the calibration/homography row, the full text of Wu et al. [10] reports two independent validation tests on a three-degree-of-freedom robot platform: a mean deviation between reconstructed and actual coordinates of 0.0196–0.0296 mm and a maximum deviation of 0.0374–0.0394 mm, measured at their own experimental pixel-to-distance scale of approximately 95.5 µm/pixel. Expressed in pixels, this corresponds to a mean-to-maximum accuracy of approximately 0.20–0.41 pixels, which we reapply at the SIDO-CDAM’s 20 µm/pixel scale below. For motion correction, Jiao et al. [13] validate the RANSAC-ESM approach through comparison plots against laser-sensor displacement data rather than a single stated numeric RMS or maximum-error value.

Two further components (lens distortion residual after correction, illumination-induced edge shift, and thermal drift after warm-up) are not numerically quantified in any of the reviewed studies for a directly comparable optical configuration and are listed as open items rather than estimated.

Combining the two quantified components as: feature localization (1.0–2.0 µm) and calibration/homography residual (4.2–8.2 µm, based on [10]’s reported data), by RSS gives a partial system-level uncertainty of approximately 4.3–8.4 µm. This already reaches or exceeds the 1–5 µm target before the remaining open uncertainty sources are included. Therefore, the target should be considered as a design goal rather than a validated result. Closing this gap requires either a numeric motion-correction residual from Jiao et al. [13] (published figures not available), experimental characterization of the remaining open uncertainty sources, or higher optical magnification; for example, 5× magnification would reduce the calibration/homography contribution to approximately 1.1–2.1 µm. Validation follows the protocol described in Section 7.2.

For the prototype-scale SIDO-CDAM developed by Ozarkar et al. [1], an overall expanded uncertainty within approximately 1–5 µm is taken as the design target sufficient to resolve experimentally observed finite element analysis (FEA) discrepancies (Table 6).

The proposed framework integrates all major stages required for synchronized, non-contact, and uncertainty-aware characterization of SIDO-CDAMs within a single experimentally implementable methodology. Table 7 gives the full specification.

### 7.1. Illustrative Prototype-Scale Estimation

For the prototype-scale SIDO-CDAM developed by Ozarkar et al. [1], having separate output ports, the proposed framework targets a measurement uncertainty of approximately 1–5 µm as a design goal, under suitable calibration and illumination conditions; this target is not yet experimentally demonstrated. A 12 MP industrial camera and a telecentric lens at 1× magnification result in a typical spatial resolution of about 20 µm/pixel. The literature-reported localization accuracy of the Zernike-based sub-pixel extraction is 0.05–0.10 pixels [16], resulting in an effective displacement contribution of approximately 1–2 µm at this scale factor. In accordance with the microscopic vision-based measurements reported by Yao et al. [23], replacing the telecentric lens with microscopic objectives (e.g., 5×–10× magnification) would reduce the µm/pixel scale factor for MEMS-scale compliant mechanisms. However, implementation at the MEMS-scale would require re-specification of marker fabrication, illumination, and vibration tolerances to account for the increased sensitivity associated with the smaller scale (Section 4.2); therefore, increased magnification alone would not be sufficient.

### 7.2. Out-of-Plane Motion, Dynamic Bandwidth, and Validation Protocol

The homography model in Layer 3 assumes strictly planar motion; therefore, out-of-plane deflection of thin flexure hinges in compliant mechanisms cannot be directly measured by the proposed single-camera 2D system framework. This is a genuine limitation. Using the working distance of 100 mm and telecentric magnification specified for the prototype-scale system, an out-of-plane displacement of approximately 10 µm may produce an apparent in-plane error of approximately 1 µm (Table 5, Gap 3), which is comparable to the feature-localization contribution in Table 6. This effect may be mitigated by monitoring the apparent size or sharpness of the tracked fiducial markers across the image sequence as an indirect defocus-based proxy for out-of-plane motion and by constraining the experimental design so that flexure hinges most susceptible to out-of-plane buckling are oriented for maximum in-plane visibility. However, true out-of-plane measurement would require an additional out-of-plane-sensitive instrument (second camera or a dedicated sensor, such as a chromatic confocal or triangulation sensor), which is outside the scope of the proposed single-camera framework and is noted as future work in Section 8.

The dynamic bandwidth characterization capability described in Section 7 also depends on camera exposure time, frame rate, illumination intensity, and trigger synchronization, and is only demonstrated in the reviewed literature for the specific conditions each study used. For the quasi-static voltage-ramp testing (<1 Hz) described in Section 4.5, the framework uses triggered acquisition, wherein each voltage step is followed by a mechanism-specific settling time before image capture to allow transient vibrations to decay. Since the damped natural period of the Ozarkar et al. [1] prototype is not reported, the settling time cannot be specified numerically and should be determined from its modal characteristics. For dynamic testing near resonance, synchronized strobe illumination noted in Layer 2 may be used to reduce motion blur, with the required frame rate and strobe timing determined by the prototype’s resonance characteristics, as demonstrated by Hagara et al. [19].

For the proposed Layer 7 validation protocol, three aspects require explicit specification beyond a laser interferometer or precision stage is used: (a) temporal synchronization, where the vision system and the reference instrument should share a common hardware trigger (e.g., the actuator voltage-step trigger driving both camera capture and interferometer sampling) rather than relying on software-timestamp alignment, which is not precise enough at the sub-second settling times involved; (b) spatial alignment, where the interferometer beam and the vision system’s tracked fiducial marker should be co-located at the same physical point on the output port, ensuring both instruments measure displacement of same physical location; and (c) combined uncertainty, where the reported validation discrepancy between the vision system and the reference instrument should be interpreted against the RSS combination of the vision system uncertainty (Table 6) and the reference instrument’s own manufacturer-stated uncertainty, rather than either uncertainty alone. These are protocol requirements that can be explicitly stated without requiring additional experimental data.

## 8. Conclusions

The reviewed literature shows that the fundamental components required for a reliable 2D vision-based characterization system have been extensively investigated. Techniques such as Zhang-based camera calibration, homography-based coordinate mapping, sub-pixel feature extraction, camera motion compensation, and vision-based measurement of compliant mechanisms have each demonstrated high accuracy in their respective applications. However, these methods have largely been studied in isolation, on different hardware and under different experimental conditions. An integrated framework that combines all of these techniques for synchronized characterization of SIDO-CDAM has not yet been reported. The specific contribution of this review beyond that combination is the synchronized dual-output tracking layer, the associated Motion Symmetry Index, and the literature-grounded uncertainty-budget methodology.

The review highlights that calibration and motion-correction residuals (Table 6) are the main open uncertainty sources, while illumination and thermal drift remain secondary, unquantified contributors that would extend this further once characterized. The reviewed, individual measurement approaches (Section 4.6) have a clear domain of suitability rather than a single approach dominating; microscopic vision [23,24] offers the best accuracy but is over-specified for the prototype scale, incremental force-compliance testing [25] integrates well with the proposed acquisition protocol, and stereo vision and DIC [6,27] are less suitable for a strictly planar mechanism. The proposed framework is particularly suited to SIDO-CDAM characterization, which rests on synchronized multi-point tracking (Layer 6) and the Motion Symmetry Index; however, this remains a design proposal and has not yet been experimentally demonstrated.

Another common observation across the literature is the mismatch between finite element predictions and experimental measurements of compliant mechanisms. These differences are generally attributed to manufacturing tolerances, material behavior, assembly conditions, and uncertainties in boundary constraints. Although vision-based measurement systems provide a flexible alternative to conventional sensors, studies focusing on the simultaneous tracking of multiple outputs for comprehensive experimental characterization remain limited.

Future research should therefore focus on integrating these established techniques into a unified vision-based measurement framework and validating its performance experimentally. Further efforts are also needed to improve robustness under dynamic operating conditions, extend the methodology to three-dimensional motion compensation, and use synchronized multi-point displacement data for finite element model updating and uncertainty analysis.

## Figures and Tables

**Table 1:** Camera calibration and planar measurement accuracy benchmarks are reported in the reviewed studies.

Study	Method	Calibration Error	Accuracy	Application
Wu et al. (2023) [10]	Homography + SVD	Sub-pixel	High accuracy & repeatability	3-DOF robot stage
Arellano-González et al. (2021) [9]	DLT calibration	Equivalent	Trajectory tracking	Four-bar mechanism
Chen et al. (2016) [11]	2D DFT phase/grating	<1 pixel	3.5 nm (X), 8 nm (Y)	Planar stage encoder
Jiao et al. (2021) [13]	RANSAC + ESM homography	Sub-pixel	<1 pixel	Structural displacement
Moru & Borro (2019) [14]	Vision2D sub-pixel	0.06 pixel	±0.020 mm	Gear inspection
Nogueira et al. (2023) [15]	Monocular sub-pixel edge	—	0.008 mm mean	Mechanical part dimensions.

**Table 2:** Overview of sub-pixel edge detection accuracy in reviewed studies.

Study	Algorithm Family	Max Error	Repeatability	Application
Xie et al. (2019) [16]	Roberts + Zernike hybrid	<2 µm	<1 µm	Industrial parts (general)
Cheng et al. (2025) [21]	Canny–Steger + Hessian	3 µm	2 µm	Shaft dimension measurement
Duan et al. (2018) [20]	Gaussian integral	1.9 µm	N/R *	Gear tooth profiles
Guo et al. (2024) [17]	Zernike matrix method	Sub-pixel (vs. CMM)	Sub-pixel	Flange disk dimensions
Hagara et al. (2024) [19]	Grey-level moment (GLM)	<2 pixels dynamic	<0.5 pixel	Vibration monitoring
Nogueira et al. (2023) [15]	Sub-pixel monocular	0.013 mm (circular)	0.006 mm	Planar part dimensions

* N/R = not reported.

**Table 3:** Comparative assessment of vision-based characterization approaches against SIDO-CDAM requirements.

Approach	Demonstrated Accuracy	Displacement Range Validated	Experimental Complexity	Suitable for SIDO CDAM
Microscopic vision, multi-scale Lucas-Kanade [23]	0.06 µm	sub-µm to µm range, Nano-positioning stage	High; high-magnification optics, narrow depth of field	High for MEMS-scale variants; over-specified and impractical (working distance, field of view) for 60 mm port separation of prototype-scale system
PSO, S-TSS template matching, microscopic [24]	sub-µm; high computational efficiency	3-DOF compliant micro-stage	Moderate; computationally lighter than full optical-flow tracking	Similar scale limitation to [23]; the template-matching approach itself, decoupled from microscopic optics
Incremental force-compliance/vision-way testing [25]	Not quantified; accuracy is inherited from underlying image-based displacement measurement, not from the loading procedure.	Demonstrated on small compliant mechanisms; range not specified	Low; standard camera, stepped loading, no specialized optics required	High; directly applicable as actuation protocol for voltage-ramp testing
Full-pose kinematic calibration [26]	Not quantified; reported as sufficient for parallel-robot parameter identification	Parallel robotic mechanism, range not specified	Moderate; single camera with mechanism-specific identifiability analysis	Moderate; identifiability concept is transferable to confirm SIDO-CDAM parameters (GA, parasitic ratio, port positions) observable from image data alone, but full-pose calibration unnecessary for planar mechanism
Stereo vision [27]	Errors below 10% vs. FEA	3D compliant gripper mechanism	High; dual-camera calibration, baseline alignment sensitivity	Low for planar SIDO-CDAM; third dimension recovered by stereo is not needed, and calibration complexity is not justified by accuracy achieved
Digital Image Correlation [6]	Not separately quantified; commercial systems (Aramis, Vic-2D) report sub-pixel field-wide accuracy in general use.	Full-field, general experimental mechanics	High; speckle preparation, high computational cost, high-magnification optics reduce field of view	Low for miniaturized flexures specifically; speckle pattern application and field-of-view constraints make marker-based tracking more practical

**Table 4:** Measurement method comparison for SIDO-CDAM characterization.

Method	Accuracy	Simultaneous DOFs	Contact	Dynamic	Suitable for SIDO CDAM
Laser interferometer	<1 nm	1 per head	No	Yes (kHz)	No—needs 6 heads for SIDO
Grating/time-grating	~1 nm	2 (X + Y)	No	Yes	Partial—requires scale attachment
CMM contact probe	~0.5 µm	3D sequential	Yes, loads	No	No—stiffness loading; quasi-static only
Strain gauge	~0.1 µm	1–3	Yes, loads	Yes (kHz)	No—alters resonance frequency
Passive CDAM meter [33]	<10 nm	3 (indirect)	No (indirect)	Limited	No—requires secondary mechanism
2D Vision (proposed)	1–5 µm	All in-plane DOFs	No	Yes (high-speed)	Yes—full SIDO characterization
Microscopic vision [23]	0.06 µm	All in-plane DOFs	No	Limited	Yes for MEMS; over-specified for prototype

DOFs = degrees of freedom; Accuracy figures are drawn from heterogeneous sources spanning different fields of view, working distances, and reference standards (Table 1 and Table 2); they are reported here as published to indicate order-of-magnitude capability per method category, not as a controlled head-to-head comparison.

**Table 5:** Prioritized research gaps in 2D vision characterization of SIDO-CDAMs.

SR	Gap	Consequence	Priority
1	No end-to-end validated framework for SIDO-CDAM characterization	No experimental validation of agreement between FEA and physical prototype response; symmetric amplification behavior not fully validated	Critical—primary contribution
2	Simultaneous dual-output measurement not demonstrated	Sequential single-point measurements cannot detect cross-port coupling and synchronized motion behavior	Critical
3	Out-of-plane flexure deflection error not quantified	Homography assumes planar motion, and 10 µm out-of-plane deformation leads to ~1 um apparent in-plane error at a working distance of 100 mm.	High
4	Dynamic testing bandwidth versus illumination trade-off unresolved	Resonance-frequency testing requires synchronized illumination or high-speed imaging; no standard CDAM implementation exists currently	High
5	Absence of a standardized validation protocol	Different studies report different error metrics (maximum error, mean error, RMS error), which makes cross-study comparison difficult.	Medium
6	FEA model updating using vision data not demonstrated	Experimental multi-point displacement data have not been fully exploited to identify dominant error sources in compliant mechanism models	Medium—further work

**Table 6:** Proposed seven-layer vision framework—applicable to both prototype and miniaturized MEMS-scale CDAMs.

Layer	Specification	Reference	Gap Addressed
1: Optics	Telecentric lens 25–50 mm (prototype) or microscopic objective (MEMS); 12–20 MP monochrome sensor; WD 100–200 mm (prototype) or 5–20 mm (MEMS)	Nogueira et al. [15]; Cheng et al. [21]; Yao et al. [23]	Gaps 1, 3
2: Illumination	Coaxial diffuse LED + current-regulated driver (<0.1% ripple); 10 min warm-up. Strobe LED for dynamic testing at resonance frequency.	Xie et al. [16]; Hagara et al. [19]	Gaps 1, 4
3: Calibration	Zhang checkerboard (≥20 poses); full distortion model; homography characteristic matrix + SVD back-projection	Wu et al. [10]; Arellano-González et al. [9]	Gaps 1, 5
4: Motion correction	Fixed reference markers on base plate; per-frame RANSAC homography drift subtraction	Jiao et al. [13]	Gaps 1, 3
5: Feature extraction	Coarse-precise Zernike hybrid (Xie et al. [16]) on output marker edges; GLM for dynamic blur conditions	Xie et al. [16]; Hagara et al. [19]	Gap 1
6: Dual-output tracking	Simultaneous: input port + Output Port 1 + Output Port 2; cross-correlation → GA + parasitic ratio + symmetry check	Ozarkar et al. [1]; Clark et al. [33]	Gap 2
7: Validation	Primary axis: laser interferometer. Parasitic axis: precision stage sweep. Report mean ± 2σ, ≥5 load cycles, quasi-static + one dynamic point.	Yao et al. [23]; Clark et al. [33]	Gaps 4, 5

**Table 7:** Literature-grounded uncertainty budget for the proposed framework (design-stage estimate, not experimentally measured on the SIDO-CDAM prototype).

Component (Layer)	Literature Value	Basis	Contribution at ~20 µm/px
Calibration + homography residual (Layer 3)	0.20–0.41 px, derived from 0.0196–0.0394 mm deviation reported at ~95.5 µm/px in [10] own experiments	Directly computed from [10]’s reported physical-unit deviations; pixel scale, converted to pixel units for reuse at the SIDO-CDAM’s scale	4.2–8.2 µm
Motion/drift compensation residual (Layer 4)	“Sub-pixel accuracy,” not quantified as a single scalar value in the extractable text of [13]	Open item; validated only via comparison plots [13]	Not estimated; bounded only by the qualitative “sub-pixel” (<1 px) [13]
Sub-pixel feature localization (Layer 5)	0.05–0.10 px [16]	Directly reported value for Zernike hybrid extraction	1.0–2.0 µm
Lens distortion (post-correction, telecentric)	Not quantified in the reviewed literature	Open item	Not estimated—flagged for design-stage, FEA analysis or experimental characterization
Illumination-induced edge shift	Not quantified in the reviewed literature	Open item	Not estimated
Thermal drift (CMOS, post warm-up)	Not quantified in the reviewed literature	Open item	Not estimated

## Data Availability

No new data were created or analyzed in this study. Data sharing is not applicable to this article.

## Abbreviations

The following abbreviations are used in this manuscript:

SIDO	Single Input Dual Output
CDAM	Compliant Displacement Amplification Mechanism
GA	Geometrical Advantage
FEA	Finite element analysis
DOF	Degrees of Freedom
DFT	Discrete Fourier Transform
DLT	Direct Linear Transformation
SVD	Singular Value Decomposition
RANSAC	Random Sample Consensus
ESM	Efficient Second-order Minimization
DIC	Digital Image Correlation
ISO-GUM	International Organization for Standardization—Guide to the Expression of Uncertainty in Measurement
